# The role of race and ethnicity in health care crowdfunding: an exploratory analysis

**DOI:** 10.1093/haschl/qxae027

**Published:** 2024-02-28

**Authors:** Sara Machado, Beatrice Perez, Irene Papanicolas

**Affiliations:** Department of Health Services, Policy and Practice, Brown University School of Public Health, Providence, RI 02903, United States; Department of Health Policy, London School of Economics, London WC2A 2AE, United Kingdom; Department of Computer Science, University of Massachusetts, Boston, MA 02125, United States; Department of Health Services, Policy and Practice, Brown University School of Public Health, Providence, RI 02903, United States; Department of Health Policy, London School of Economics, London WC2A 2AE, United Kingdom; Department of Health Policy and Management, Harvard T.H. Chan School of Public Health, Boston, MA 02115, United States

**Keywords:** racial disparities, medical crowdfunding, access to care, organ transplantation

## Abstract

Medical crowdfunding is a key source of financing for individuals facing high out-of-pocket costs, including organ-transplant candidates. However, little is known about racial disparities in campaigning activity and outcomes, or how these relate to access to care. In this exploratory, nationwide, cross-sectional study, we examined racial disparities in campaigning activity across states and the association between US campaigners’ race and ethnicity and crowdfunding outcomes using a novel database of organ-transplant–related campaigns, and an algorithm to identify race and ethnicity based on name and geographic location. This analysis suggests that there are racial disparities in individuals’ ability to successfully raise requested funds, with Black and Hispanic campaigners fundraising lower amounts and less likely to achieve their monetary goals. We also found that crowdfunding among White, Black, and Hispanic populations exhibits different patterns of activity at the state level, and in relation to race-specific uninsurance and waitlist additions, highlighting potential differences in fundraising need across the 3 groups. Policy efforts should consider not only how inequalities in fundraising ability for associated costs influence accessibility to care but also how to identify clinical need among minorities.

## Teaser

Organ-transplant candidates facing high out-of-pocket health expenditures may turn to medical crowdfunding for financial support, although racial disparities may hinder minority campaign organizers, particularly Black and Hispanic individuals, in their efforts to successfully raise requested funds compared with their White counterparts. In an exploratory analysis using US-based crowdfunding campaigns, after accounting for crowdfunding campaign and regional characteristics, we found that Black and Hispanic campaigners fundraise 16% and 21% lower amounts and were 13% and 18% less likely to achieve their monetary goals. By examining crowdfunding activity in relation to uninsurance rates and waitlist additions at the state level we found that crowdfunding among White, Black, and Hispanic populations exhibits different patterns of activity at the state level, highlighting potential differences in fundraising need across the 3 groups. Policymakers cannot resort to a one-size-fits-all approach when attempting to improve access to care for transplant patients.

## Introduction

Crowdfunding has become a common source of financing for those struggling to cover health care costs, even among individuals with health insurance, due to substantial out-of-pocket expenses, including deductibles, co-payments, or even travel expenses.^1-4^ As a result, the health care industry has dubbed crowdfunding as a “digital safety net,” signaling its potential to offer financial coverage to under- or uninsured individuals.^5^ Prior literature has shown that the size and financial liquidity of campaigners’ social networks are critical factors for fundraising success.^2,3,6-8^ Yet, these factors may potentially reinforce, and even exacerbate, existing social and racial inequities in health care access and coverage as crowdfunding becomes more widespread.^6,9-11^

Racial and ethnic disparities have been well documented across federal, state, and local data, and exist in relation to health outcomes, utilization, and coverage.^12-15^ Historically, racial and ethnic minorities are more likely to be uninsured and encounter greater barriers to accessing health care, as compared with White individuals.^16^ While the existence of crowdfunding platforms (CFPs) may offer an opportunity to overcome these barriers to access, evidence shows that not all campaigns reach their monetary goal^3,11^ and that crowdfunding favors individuals with larger social networks of more affluent individuals, as well as those who are able to engage with online tools and tell a compelling story.^3,6,10,17-20^ Additionally, the link between crowdfunding and health insurance is not clear. One would expect to see more crowdfunding in geographical areas with lower insurance or underinsurance rates among populations with similar levels of clinical need; however, there is mixed evidence on the association between Medicaid expansion under the Affordable Care Act (ACA) and higher levels of medical crowdfunding.^3,11^ Further evidence suggests that underinsured individuals do not receive higher donation amounts.^7,11,21^ More work is needed to better understand whether crowdfunding outcomes and their relationship with health insurance coverage differ significantly by race and ethnicity and, thus, whether this form of health care financing further widens existing racial disparities.

In this paper, we examine racial and ethnic disparities in crowdfunding for transplant-related costs, since the ability to afford the full range of transplant-related expenditures can pose a substantial barrier to care^17,22,23^—namely, by preventing access to a solid-organ transplantation waiting list.^15,22,23^ Concurrently, racial and ethnic minorities face higher waiting times for transplantation, higher waitlist mortality, lower transplantation rates, and worse clinical outcomes.^15,24-26^ Using a unique database comprising a cross-section of 19 711 US-based crowdfunding campaigns related to transplantation, available online in 2019 and from 7 CFPs, we explored 3 main questions: (1) How does crowdfunding vary by campaigner race and ethnicity, (2) What is the association between crowdfunding outcomes and campaigners’ race and ethnicity, and finally, (3) How much of this variation is due to regional disparities in clinical need and insurance coverage?

## Data and methods

### Campaign identification

We first identified all US-based CFPs with individual medical crowdfunding campaigns available online in March 2019 (*n* = 7). In Table S1 we outline details on exclusion criteria of identified CFPs not included in the study, which range from business CFPs (*n* = 7), CFPs for charities (*n* = 2), non–US-based CFPs (*n* = 2) or CFPs in which it was not possible to automate the search criteria (*n* = 2). Potentially eligible campaigns were detected within each CFP using each CFP's internal search engine. We first identified and collected 55 252 unique URLs using a systematic search strategy with the terms “organ transplant” (where organ was 1 of kidney, liver, heart, lung, pancreas, intestine, and bone marrow). We fine-tuned the search strategy using a geographic query whenever the number of search results exceeded those made available by the CFPs. This geographically stratified query was performed using the search terms “‘organ’ transplant in ‘location’”. Location is defined as a city, state as identified in the US Cities Database (data sources: US Census Bureau, US Geological Survey, and American Community Survey), which also contains geocoding, county, and population information. The search algorithm was optimized across the 44 532 cities in the database to ensure a wide coverage of the US territory. To do so, we ran separate and overlapping queries across 3 subsets of cities, defined as (1) cities in which there was at least 1 Centers for Medicare and Medicaid Services (CMS) provider hospital (1993–2017; data source: CMS Hospital Data [from Provider of Services]), (2) the 25 largest population centers per state, and (3) geographical clustering centered around self-reported campaign location, which casts a net around each city in which we have identified at least 1 campaign (source algorithm: density-based spatial clustering of applications with noise [DBSCAN]). Figure S1 shows the geographic coverage of each query and the overall combined coverage. Duplicate campaign search results were then discarded.

### Data extraction and campaign selection

To collect the information contained in the campaign database identified above we created a web crawler, similar to the one we used to develop our fraud detection algorithm,^27^ and tailored it to each of the 7 CFPs hosting charitable fundraisers (GoFundMe, Fundly, YouCaring, GiveForward, MightyCause, Fundrazr, and Indiegogo). We scraped data from 41 565 of those campaigns, including information on donation amounts, number of donors, campaign organizer and beneficiary, and self-reported geographic location.

### Exclusion criteria

Exclusion criteria were as follows: (1) identification as not US-based, (2) invalid data-scraping output for any of the variables (eg, text where numeric output was expected), (3) not including the word “transplant” in the story, and (4) outliers in amount raised and/or number of donors (Figure S2).

### Variable identification: organ type, state, and date of creation

To categorize campaigns according to organ type, bigram analysis was performed on the campaign's story. First, text was pre-processed by (1) lower capitalizing all text, (2) removing all stop words (frequently used, yet meaningless, words, such as prepositions and helping verbs), and (3) removing all punctuation. Second, for each organ and across all campaigns, all pairs of words of the type before (ie, “word + organ”) or after (ie, “organ + word”) were collected. Third, the number of times each pair was present across all stories was counted. Fourth, word pairs were ranked according to their frequency, separately for before and after pairs, by organ. Fifth, organ-specific and clinically relevant words were selected, as well as those indicating need for organ transplant (Table S2). Finally, campaign was categorized as “organ”-specific if it included any clinically relevant words adjacent to “organ” (either before or after word pairs). Campaigns classified as specific to more than 1 organ were labeled as multiple. Campaign organ type was otherwise set to missing.

Direct analysis of scraping output allowed us to categorize campaigns into (1) not in United States, (2) locations in United States (city, state matched with US cities list), and (3) incorrect. Incorrect campaigns’ state information was completed by analyzing each campaign's story, when possible. State information was completed (1) if the story explicitly mentioned the city and/or state of campaign host or (2) if the story explicitly mentioned the city and/or state of campaign beneficiary (if the host location was not present and host not the beneficiary). Campaign state was otherwise set to missing. The campaign date of creation was directly available from scraping output or otherwise set to missing.

We had a final sample of 19 711 campaigns created between 2015 and 2019 with information on campaign organizer name, geographic location, and fundraiser organ type.

### Data quality verification procedures

To ensure accuracy and quality of data collection, 350 campaigns were manually and independently verified by 2 authors (S.M. and B.P.). A quality check targeted whether output from the data-extraction tool complied with the search criteria and campaign inclusion requirements.

### Crowdfunding campaign outcomes and characteristics

We obtained information on the amount raised (in US dollars), the number of donations, and the monetary goal directly from the campaign data. We used these metrics to define the 3 crowdfunding outcomes: (1) amount raised (in US dollars), (2) success rate (the ratio between amount raised and the monetary goal posted on the campaign), and (3) average donation (the ratio between amount raised and number of donations). We also directly extracted or computed a set of campaign characteristics based on the available data. These included the year of the campaign's creation and the number of social media shares (Facebook shares). We created a dummy variable identifying the campaigns in which the campaign organizer is not the beneficiary and computed each campaign's fraud score using a crowdfunding fraud-detection algorithm.^27^ Finally, we used a natural language processing (NLP) algorithm using word vectors and bi-gram analysis to classify each campaign according to the type of organ it was fundraising for, as described above.

### National- and state-level variables

To measure the clinical need for transplantation, we extracted data on yearly state-level number of waiting-list additions (WLAs), by race and ethnicity and between 2015 and 2019, from the United Network for Organ Sharing (UNOS) database (data available at https://optn.transplant.hrsa.gov/data/). We collected yearly state uninsurance rate, by 100 000 race/ethnicity-specific population between 2015 and 2019, from the Kaiser Family Foundation (KFF) state profiles to measure financial need (see https://www.kff.org/statedata/). These estimates are based on the American Community Survey 1-year sample. Information on Medicaid expansion status was collected from the KFF State Medicaid Facts (see https://www.kff.org/state-category/medicaid-chip/) and current Population Survey estimates of race/ethnicity population between 2015 and 2019 were obtained from Census.gov.

### Methods

#### Crowdfunding campaigner race and ethnicity classification

We assigned 1 of 4 race/ethnicity categories to the crowdfunding campaign organizer using the NAMSOR classifier: White non-Hispanic, Black non-Hispanic, Hispanic, and Other non-Hispanic.^28,29^ Onomastic classifiers are increasingly used to characterize individuals’ race and ethnicity (and potentially gender) when the information is not directly available from the subject.^30-32^ We use NAMSOR API, a commercially available classifier (https://namsor.app/), as it computes the probability of the 2 most likely race/ethnicity pairs using 3 elements—first name, last name, and zip code—and it has been evaluated as having high accuracy among similar tools.^33^ The race/ethnicity categories map onto the classifications provided by the Centers for Disease Control and Prevention (CDC) WONDER database.

The geographical element is important to improve accuracy and harnesses the geographic information in our data. The first and last names of the campaign organizer were collected directly from the campaign data, and the campaign organizer's self-reported “city, state” was used to assign the relevant zip code(s) information using the US Cities List database.^34^ We ran the NAMSOR classifier for all “first name, last name, zip code” sets in the data and recorded the estimated probability for the most likely race/ethnicity for each campaign. We assigned a single race/ethnicity category to each campaign organizer, based on their highest estimated probability. If, based on a set of zip codes, a campaign was assigned more than 1 possible race/ethnicity (*n* = 1354, 6.9% of the campaigns), we computed the race/ethnicity score as a weighted average of the estimated race/ethnicity probabilities identified by NAMSOR. For example, consider 1 campaigner who was assigned 10 possible zip codes, 7 of which identifying “White” as the most likely race/ethnicity: their score is the weighted average of 7 “White” estimated probabilities, where the weight is the proportion of “White” outcomes, in this case 7 of 10.

As this could potentially introduce a bias in the classification, we conducted a sensitivity analysis using an alternative classification procedure that simply chooses the race/ethnicity according to the highest estimated probability across all possible zip codes to assess whether alternative procedures led to substantial classification disparities, considering the limitation of dealing with classifiers with less than 100% certainty (see Supplementary Analysis including Supplementary Figure 3-6 and Supplementary Table 9 for the results).

#### Campaign activity and WLAs by race and ethnicity

To examine differences in fundraising activity across race and ethnicity, we computed the proportion of total and solid-organ (heart, lung, kidney, liver, pancreas) transplantation campaigns by race and ethnicity for the study period (2015–2019). (Since campaign data were available until March 2019, we used linear interpolation to attribute 25% of 2019's waiting list additions to the study period.) We computed total WLAs by race and ethnicity for the same set of solid organs over the same period.

#### Campaign characteristics and outcomes by race and ethnicity

We produced mean, standard deviation (SD), median, and interquartile range (IQR) estimates on the following campaign characteristics by race and ethnicity: number of social media shares, number of campaigns that had a beneficiary other than the campaign organizer, and proportion of campaigns flagged as potentially fraudulent. Mean differences across race and ethnicity groups were tested using a chi-square *t*-test for the difference in means.

We examined the difference in performance of campaigns by race and ethnicity by the 4 outcomes described above (amount raised, number of donations, success rate, and average donation). For each outcome we computed the mean, SD, median, and IQR tested for differences across race and ethnicity groups using a chi-square *t*-test for the difference in means.

#### Association between state-level crowdfunding activity and fundraising need by race and ethnicity

We analyzed racial and ethnic disparities in the relationship between crowdfunding activity and clinical need, estimating linear ordinary square regression and the corresponding slope coefficients between states’ campaigns per 100 000 race/ethnicity population and WLAs per 100 000 race/ethnicity population. We restricted this analysis to solid-organ campaign activity by White, Black, and Hispanic campaign organizers to match what is available from UNOS. Similarly, we fitted regression lines and estimated the slope coefficients between states’ campaigns per 100 000 and uninsurance rate per 100 000 by race and ethnicity to quantify the relationship between financial need and fundraising activity.

#### Association between individual-level crowdfunding campaign outcomes and race and ethnicity

We estimated the association between crowdfunding outcomes and race and ethnicity as incidence rate ratios (IRRs) estimated by count data models. For each model, we used either negative binomial or Poisson regression according to the overdispersion of the outcome. We estimated the same model for each outcome (amount raised, success rate, average donation). The main specification included a categorical variable for race and ethnicity as our main variable of interest, using White as the comparator group (vs Black, Hispanic, or Other). We adjusted the models, including the following campaign characteristics: year of campaign creation, social media shares, an indicator for campaigns with organizer different from the beneficiary, an indicator for high probability of fraud, and an indicator for solid-organ fundraisers. We also adjusted the models for state-level characteristics, including an indicator for states in the fourth quartile of WLAs per 100 000, an indicator for states in the fourth quartile of uninsurance rate, and an indicator for states that adopted Medicaid expansion since 2014. We performed 4 sensitivity analyses: (1) restricting the sample to solid-organ campaigns, (2) excluding 2019, (3) restricting to campaigns with the same race/ethnicity classification across classification approaches, and (4) restricting this sample to campaigns above a 60% certainty threshold in the probability of race/ethnicity attribution, to account for potential bias induced by low certainty in the classification algorithm. Robust standard errors were computed and adjusted for 51 state clusters.

## Results

### Campaign activity and WLAs by race and ethnicity

Our cross-sectional sample included 19 711 campaigns created between January 2015 and March 2019 (Table 1). White campaign organizers accounted for 52% of all campaigns, Black campaigners for 17%, Hispanic campaigners for 15%, and other campaigners for 16%. The race and ethnicity proportions were similar when restricted to solid-organ campaigns (*n* = 11 998 or 61% of the campaigns; White: 51%; Black: 18%; Hispanic: 15%; Other: 16%). The WLAs were of similar proportions to campaigns for White (52%) and Hispanic (17%) campaigners. As compared with the proportion of campaigns, there was a higher proportion of WLAs for Black patients (23%) and a lower proportion for patients from other race and ethnicities (8%) (Table 1).

**Table 1.** Campaigns, campaign outcomes, and waiting-list additions, by race/ethnicity.

	Overall		White		Black		Hispanic		Other		Test, *P* value
			*n*	%	*n*	%	*n*	%	*n*	%	
Campaigns, *n*	19 711		10 295	52%	3406	17%	2924	15%	3086	16%	
Solid organs											
Campaigns, *n*	11 998		6096	51%	2180	18%	1809	15%	1913	16%	
Waiting-list additions, *n*	235 701		123 367	52%	53 893	23%	39 604	17%	18 839	8%	
										
	**Median**	**IQR**	**Median**	**IQR**	**Median**	**IQR**	**Median**	**IQR**	**Median**	**IQR**	
Crowdfunding outcomes											
Amount raised, $	2770	1270–6030	3050	1525–6480	2343	870–4950	2470	1025–5332	2785	1235–6365	<.001
Number of donations	32	15–64	34	16–66	29	12–56	31	14–60	33	14–68	.003
Monetary goal, $	10 000	5000–25 000	10 000	5000–25 000	10 000	5000–25 000	10 000	5000–25 000	10 000	5000–30 000	<.001
Success rate	27.1%	9%–59%	31.0%	11%–64%	22.4%	7%–52%	23.0%	7%–52%	24.3%	7%–56%	<.001
Average donation, $	81.9	59–113	85.8	63–118	75.5	53–106	75.0	54–104	81.7	58–113	.4

Abbreviation: IQR, interquartile range.

### Association between state-level crowdfunding activity and fundraising need by race and ethnicity

**Figure 1.**
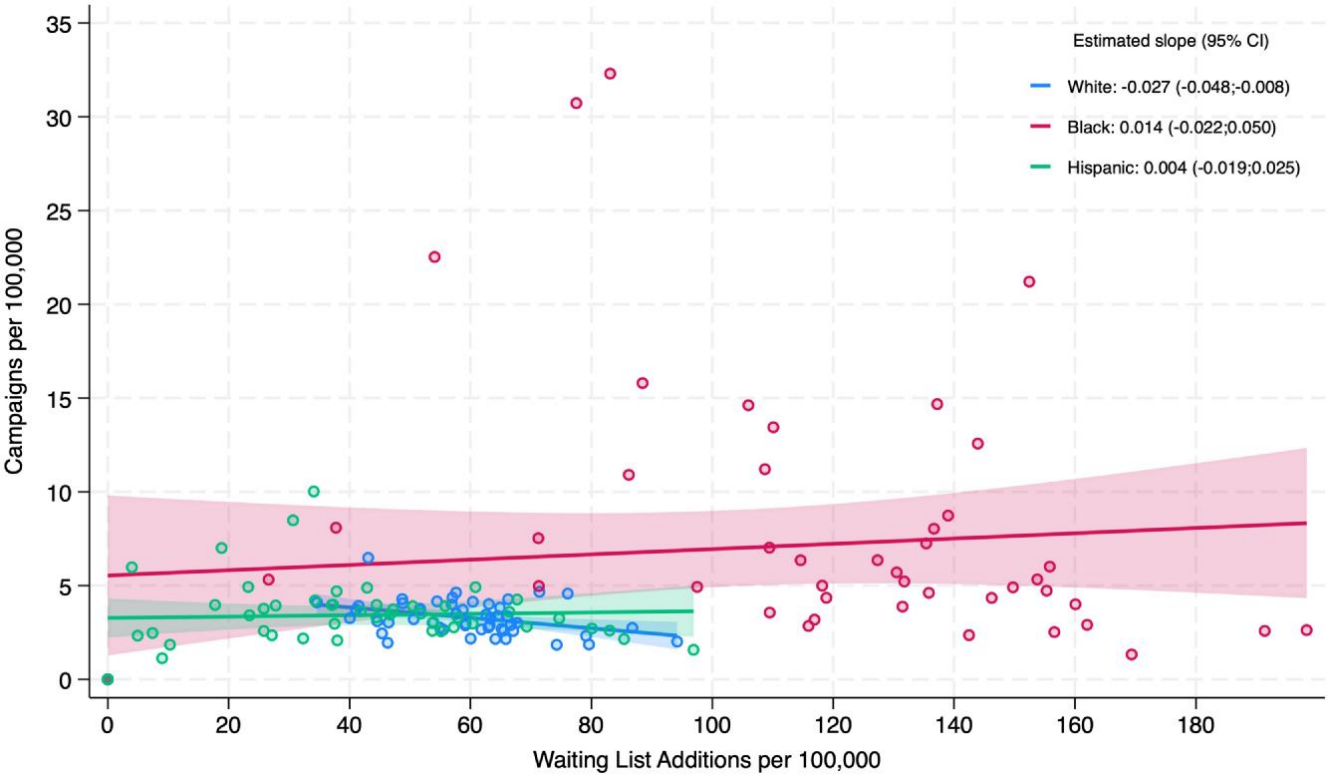
Campaigns and WLAs of solid organs (including heart, lungs, kidney, liver) per 100 000 race and ethnicity population. Includes linear fit lines per race and ethnicity, as well as the estimated slope and respective confidence intervals. To allow for sample consistency with Figure 2, excludes state-year pairs for which the uninsurance rate is not available due to small population size, according to KFF state data criteria. Abbreviations: CI, confidence interval; KFF, Kaiser Family Foundation; WLA, waiting-list addition.

At the state level, Blacks had both a higher level of campaigns and WLAs per 100 000 (Figure 1). (For state comparisons, we restricted the sample to pairs of state and race/ethnicity with a sufficient population to compute the uninsurance rate per 100 000. As such, information was not available for Blacks from Alaska, Hawaii, Idaho, Maine, Montana, New Hampshire, Vermont, and Wyoming or for Hispanics in Maine, North Dakota, and Vermont.) Black campaigners had a median of 5.33 campaigns per 100 000, as compared with 3.21 for Whites and 3.43 for Hispanics. Black campaigners also had much higher median WLAs per 100 000 (122.6) as compared with Hispanics (39.18) and Whites (57.6). The estimated slope of the linear regression of campaigns per 100 000 on WLAs per 100 000 was negative and significant for White campaigners (−0.027, *P* = .008) and positive and not significant for Black (0.014, *P* = .440) and Hispanic (0.004, *P* = .740) campaigners.

**Figure 2.**
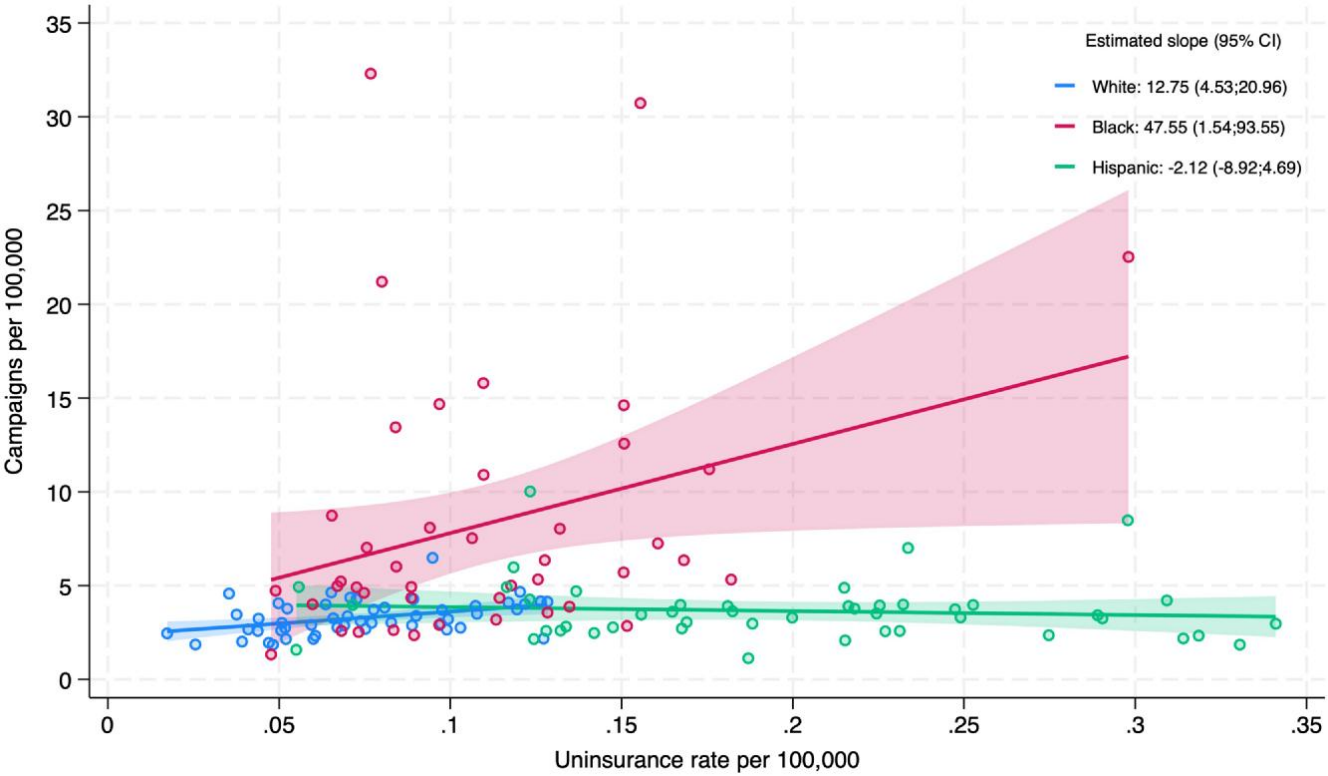
Campaigns and uninsurance rate per 100 000 race and ethnicity population. Includes linear fit lines per race and ethnicity, as well as the estimated slope and respective confidence intervals. Excludes state-year pairs for which the uninsurance rate is not available due to small population size, according to KFF state data criteria. Abbreviations: CI, confidence interval; KFF, Kaiser Family Foundation.

At the state level, median uninsurance rates per 100 000 were highest for Hispanics (0.19) as compared with Whites (0.07) and Blacks (0.10) (Figure 2). The estimated slope of the linear regression of campaigns per 100 000 on the uninsurance rate per 100 000 was positive and significant for White (12.75, *P* = .003) and Black (47.55, *P* = .043) campaigners and negative and not significant for Hispanics (−2.12, *P* = .534).

### Campaign outcomes by race and ethnicity

The median amount raised per campaign was highest for White campaigners ($3050; compared with Black, $2343, and Hispanic, $2470) (Table 1). Whites also had a higher median number of donations (34, compared with Black, 29, and Hispanic, 31), a higher success rate (31.0%, compared with Black, 22.4%, and Hispanic, 23.0), and a higher average donation amount ($85.8, compared with Black, $75.5, and Hispanic: $75.0). The test of equality of means for each outcome variable indicated that there are race/ethnicity differences in means in amount raised, success rate, and number of donations (*P* < .01). For amount raised per donor (*P* = .40), we did not reject the null hypothesis of no difference in means at the 10% significance level.

### Association between individual-level crowdfunding campaign outcomes and race and ethnicity

**Figure 3.**
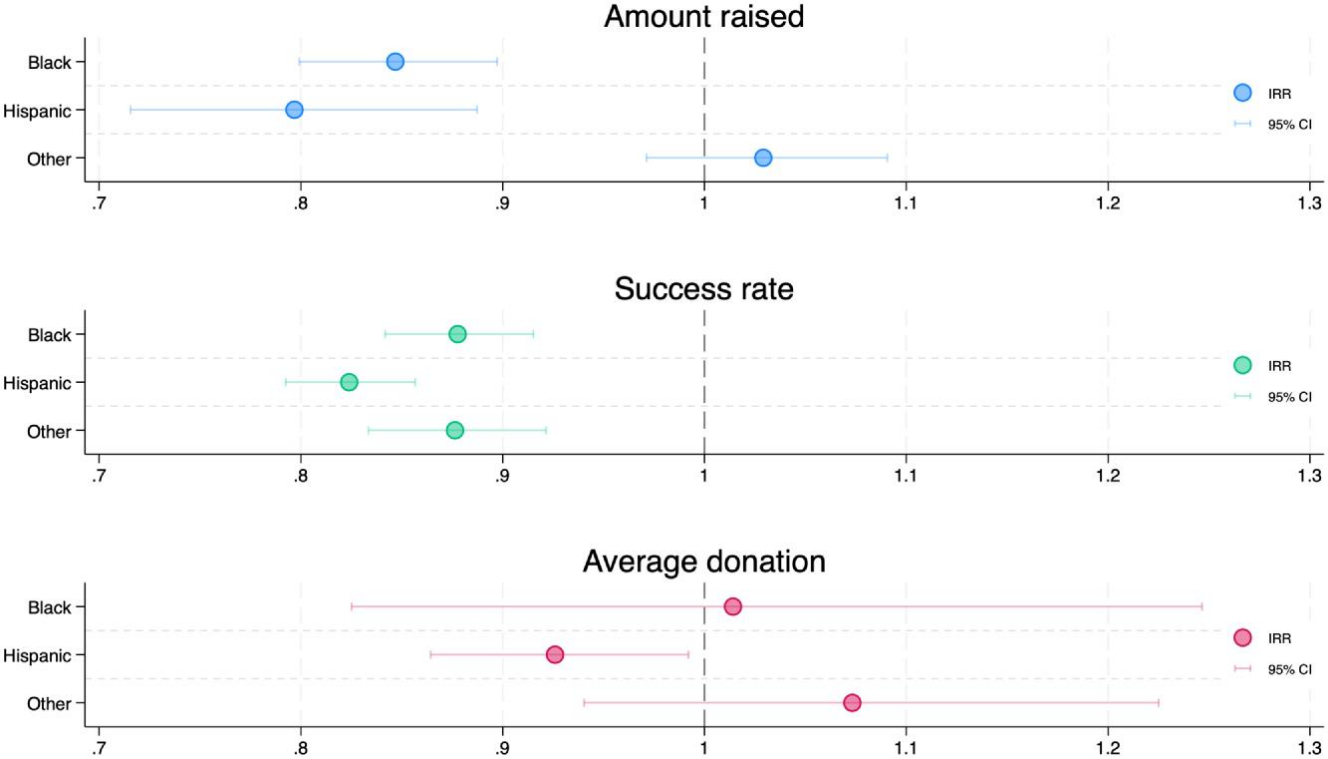
Estimates of race- and ethnicity-adjusted IRR for each crowdfunding outcome. Amount raised and average donation models were estimated using negative binomial regressions, and success rate using Poisson regression. Estimated race-specific adjusted IRRs are shown with 95% confidence intervals, with White campaign organizer as the baseline. All adjusted models include (1) dummy variables for each year of creation (baseline: 2015); (2) campaign characteristics (social media shares [including a quadratic term], a dummy variable for a campaign organizer different from the beneficiary, a dummy variable for high fraud score, and dummy variable for solid organs); and (3) state characteristics (a dummy variable for fourth quartile of race-specific WLA per 100 000 and a dummy variable for fourth quartile of race-specific uninsurance rate). Abbreviations: CI, confidence interval; IRR, incidence rate ratio; WLA, waiting-list addition.

In multivariate, negative, binomial regression analyses, campaigns organized by Black and Hispanic individuals had worse outcomes in terms of all 3 measures compared with White campaign organizers (Figure 3). Both Black (adjusted IRR: 0.847; *P* < .001) and Hispanic (0.797, *P* < .001) organizers were associated with a lower amount raised, as compared with Whites after adjustment for year, campaign characteristics, and state characteristics (Table S3). Similar effects were estimated regarding success rates for Blacks (0.878, *P* < .001) and Hispanics (0.824, *P* < .001) as compared with Whites. The average donation was only significantly lower for campaigns with Hispanic organizers as compared with Whites (0.926, *P* = .029; Blacks: 1.014, *P* = .894). The results by race/ethnicity were qualitatively similar, as well as in magnitude, when (1) computing the models with race and year alone and with race, year, and campaign characteristics alone (Table S4); (2) restricting the sample to 2015–2018 (Table S5); and restricting the sample solid-organ campaigns (Table S6). Furthermore, the results were also similar when conducting sensitivity analysis across 2 potential classification procedures (Table S7) and restricting the sample to campaigns compliant with a higher certainty threshold (*probability* > .6) (Table S8). The probability distribution by race and ethnicity and the full set of results from the alternative classification procedure are available in the Supplementary Analysis (Figures S3–S6, Table S9).

## Discussion

In our nationwide sample of crowdfunding campaigns related to organ transplantation, we found that the majority of fundraising for transplantation was organized by White campaigners, who performed better in terms of amount raised and success in meeting crowdfunding goals.

Black and Hispanic race and ethnicity were associated with lower amounts raised and a lower success rate in achieving the campaign's stated monetary goal when compared with White campaign organizers and adjusted for campaign and state characteristics. White populations are more likely to campaign in states with lower WLAs and high race-specific uninsurance rates. Black campaigners are more likely to organize campaigns in states with higher uninsurance rates, independently of local WLA rates. While the overall proportion of campaigns organized by Hispanics mirrors their proportion of WLAs, nationally we do not observe greater crowdfunding activity in states with higher per capita WLAs or higher uninsurance rates. Taken together, these results suggest that racial and ethnic disparities in health care coverage and access are possibly exacerbated by crowdfunding, particularly among Black patients.

Our results have important implications for policymakers interested in understanding disparities in financial coverage for transplant candidates, and the role that crowdfunding can play in bridging these. Crowdfunding among White, Black, and Hispanic populations exhibit different patterns of activity at the state level, highlighting potential differences in fundraising need across the 3 groups. Black campaigners were disproportionately more likely to have greater WLAs as compared with campaigners of other races and ethnicities. Hispanic campaigners had low WLA rates, which were not correlated with campaign activity. This lack of association could possibly indicate wider disparities in access to health care, preventing Hispanic patients from being diagnosed or even having access to health care and potentially leading to organ transplantation.

Crowdfunding data reflect financial need at all stages of the transplant journey and encompass financial needs beyond medical care (eg, travel, family accommodation, carer support). As such, it can be an important source of data to observe disparities in access to transplantation related to financial need. To be eligible for transplantation, patients in need are often required to prove financial ability to support the costs of transplant candidacy, recovery, and lifelong care. Some of these costs may be covered by insurance, but for low-income patients without insurance or the underinsured, this cost is likely to fall entirely on the individual. These costs may ultimately serve as a barrier to transplantation itself, which is corroborated by our findings. The lower fundraising activity among Hispanic populations, per capita, is not associated with either their larger state uninsurance rates or with their lower WLAs when compared with White and even Black populations. Furthermore, we found a positive association between uninsurance and fundraising activity among Black populations. As other work has shown, this financial barrier is more common among racial and ethnic minorities.^35^

Despite this, our findings do not indicate a greater representation of crowdfunding campaigns for Black and Hispanic populations. Instead, we observed similar campaigning activity by race/ethnicity in proportion to WLAs. This finding can be interpreted in 2 ways: either patients of all demographics require extra funds to support their transplantation journey or most patients resort to crowdfunding once they have been added to the waiting list. More work is needed to understand in detail the financial goals of different campaigns to see if there are systematic differences in funding objectives across different racial and ethnic groups and how they relate to the care-seeking journey. Further work exploring patient narratives on crowdfunding campaigns can provide additional insights into the specific financial needs of patients.

Our results also reinforce the notion that increased insurance coverage may contribute to mitigating disparities in financial protection for transplantation. Our observed racial disparities in crowdfunding outcomes are associated with higher uninsurance and are worse in states that did not adopt Medicaid expansion, which is in line with previous findings regarding the regional distribution of general medical crowdfunding.^36^ While increased insurance is protective overall, our results suggest that minority populations are less protected relative to their White counterparts in Medicaid expansion states. This may be related to the well-documented racial and ethnic disparities in Medicaid expansion.^11,37,38^ Further research examining campaign narratives can reveal more details of what costs campaigners are fundraising for and help further understand the coverage gap.

Importantly, our findings are in line with the literature documenting disparities in campaign success across a range of diseases or medical conditions—from organ transplantation to diabetes,^39^ cancer,^8,21^ hepatitis C,^20^ or neurological disorders^40^—either in terms of amount raised or campaigners’ ability to fundraise their stated monetary goal. Furthermore, we add to the literature suggesting that the difference in total amount raised seems to relate to the breadth of the donor pool, given the lack of evidence suggesting disparities in average donations.^11^ Perhaps unsurprisingly, it appears that crowdfunding exacerbates existing social inequalities, as it relies primarily on fundraisers’ ability to access funds from their social contacts.

This paper makes several contributions to the literature. The role of medical crowdfunding in the widening of social and racial disparities is well documented, particularly through limited fundraising success.^6,7,10,11^ We add to this literature with further evidence on widening racial disparities in medical fundraising based on clinical need and ability, particularly among Black organizers, using a comprehensive set of national crowdfunding platforms. Our data focus on a specific type of medical fundraiser, organ transplantation, which allows us to define a measure of fundraising need based on a clearly defined metric of medical need (WLAs), which is challenging when analyzing medical crowdfunding in general. This builds on data used in previous research, which have either relied on convenience samples or, most recently, dealt with the technical challenges in obtaining these data and classifying them by disease.^36,39,41^ Our data-scraping tool enabled us to conduct a systematic search for these campaigns and to create a comprehensive snapshot of targeted disease campaigns available online in a specific point in time. Combined with our campaign disease classification algorithm, this mechanism allows us to contribute to a growing literature on overcoming challenges in identifying clinical characteristics of crowdfunding campaigns.^36,41^ This paper also contributes to the growing literature documenting racial and ethnic disparities in coverage and access to care and their role in the demand for crowdfunding.^8^ Our findings are in line with previous research showing a positive impact of Medicaid expansion on access to care and increased financial protection.^11,37,38^

While our results are focused on the US population, crowdfunding is a tool that has been used to raise funding for health care in many other countries.^4^ Further work using the tools explored in this study to generate a sample of campaigns and to apply them to different demographic groups could be applied in other countries to further understand how crowdfunding interacts with other determinants of health disparity. Importantly, further work is needed to better understand how crowdfunding interacts with different health system designs, and if it is less likely to amplify disparities in systems that have more generous and equitable health system coverage.

This study is not without limitations. First, while our novel data-scraping tool enabled us to conduct a systematic search for crowdfunding campaigns and create a comprehensive snapshot of campaigns available online, our final sample size was limited by campaigns excluded due to incomplete information. This could influence the representativeness of our sample if certain populations are more likely to leave out important information. However, this limitation exists in any sample of crowdfunding data. Moreover, our approach has yielded a greater and more comprehensive sample size as compared with other studies.^3,6-8^ Second, using crowdfunding data to assess the role of race and ethnicity in health care crowdfunding is, in itself, a limitation, as the campaigns available online at any point in time do not provide a comprehensive picture of the crowdfunding needs nor the overall financial distress of patients. If there are systematic biases in what campaigns are created and kept online and if that is related to race and ethnicity, our results would not be able to isolate those components. Third, we relied on an algorithm to estimate race/ethnicity using name and location, which may result in some error attributing individual campaigns to a particular race/ethnicity. Fourth, campaigns are classified by organ type, but we were not able to identify whether the patient had already been added to a waiting list and/or received a transplant, thus making it difficult to determine at which stage of the care-seeking process an individual was seeking external funding and whether this varied by race/ethnicity.

## Conclusion

Taken together, our exploratory results suggest that crowdfunding may reinforce existing racial and ethnic disparities in the financing of transplant care. This is most likely because crowdfunding relies heavily on the means of one's social network to raise funds. Our results suggest that more generous insurance coverage can help decrease the need for crowdfunding, but we also found disparity in the success of crowdfunding across racial and ethnic groups. As such, we should not rely on crowdfunding to mitigate disparities in transplantation access. Instead, policymakers would benefit from considering how best to address inequalities in insurance coverage and underinsurance, thus promoting more equitable access to transplant waiting lists.

## Supplementary Material

qxae027_Supplementary_Data
