# Supplementary material for: The role of race and ethnicity in health care crowdfunding: an exploratory analysis
Source: Health Aff Sch. 2024 Feb 28;2(3):qxae027. doi: 10.1093/haschl/qxae027 (PMC10986198; doi:10.1093/haschl/qxae027)
Supplement: qxae027_Supplementary_Data [file qxae027_supplementary_data.zip › Supplement_02142024.docx]

**Supplement**

**Supplementary Table 1. Crowdfunding platforms analyzed.**

**Supplementary Figure 1. Locations searched by type of geographic query.**

**Supplementary Figure 2. Campaign selection flow diagram**


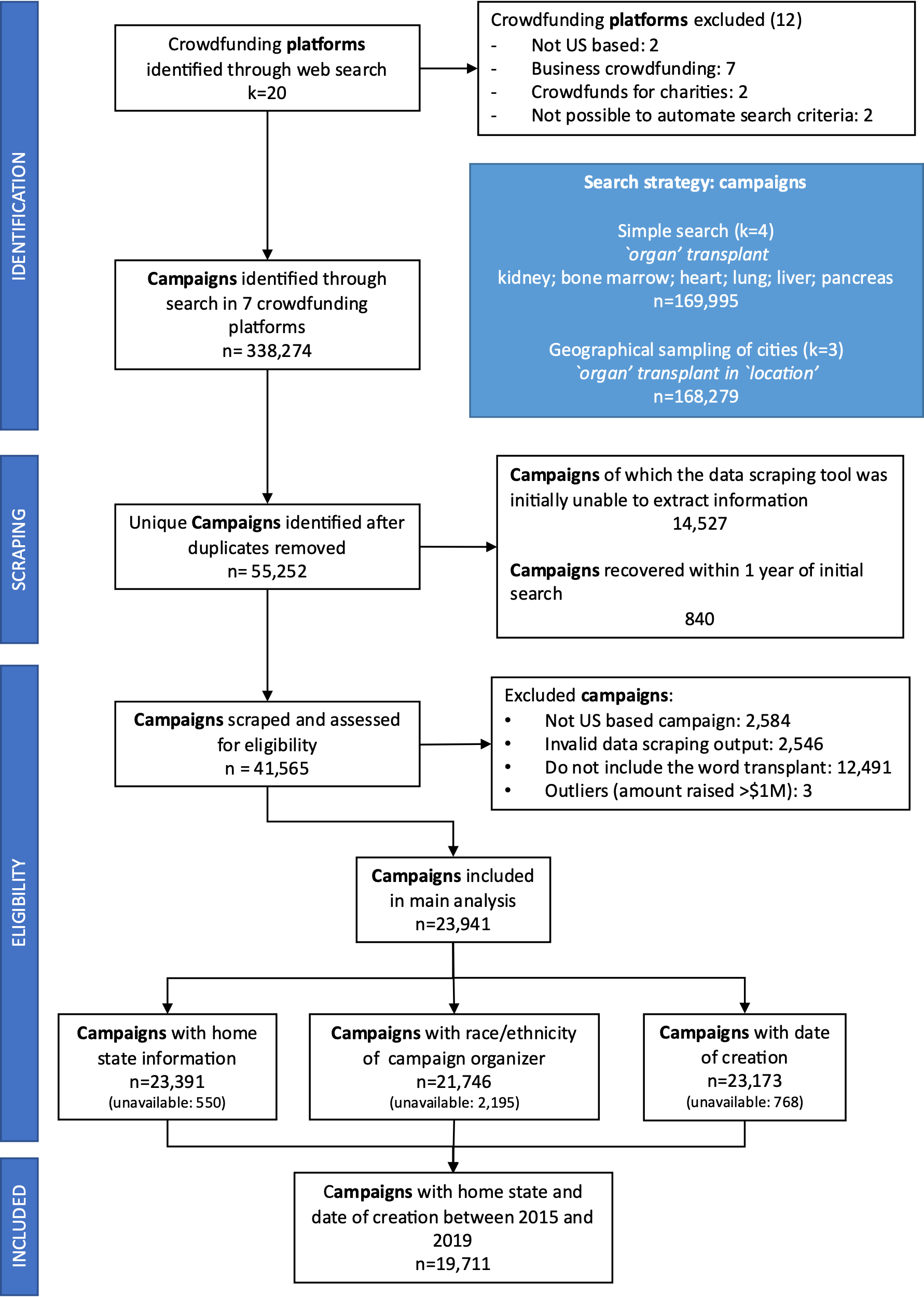


**Supplementary Table 2. Frequency of adjacent words in the classification of campaigns by organ type.**

**Supplementary Table 3. Negative binomial regression estimates - IRR for all crowdfunding outcomes - IRR and 95% CI**

|  | **Crowdfunding outcomes** | | | | | | | |  |
| --- | --- | --- | --- | --- | --- | --- | --- | --- | --- |
|  | **Amount raised** | | | **Success rate** | | | **Average donation** | | |
|  | IRR | [95% CI] | p-value | IRR | 95% CI | p-value | IRR | 95% CI | p-value |
| **Race and ethnicity** |  |  |  |  |  |  |  |  |  |
| *Reference: White* |  |  |  |  |  |  |  |  |  |
| **Black** | **0.847** | [0.799-0.897] | 0.000 | **0.878** | [0.842-0.915] | 0.000 | **1.014** | [0.825-1.246] | 0.894 |
| **Hispanic** | **0.797** | [0.716-0.887] | 0.000 | **0.824** | [0.792-0.857] | 0.000 | **0.926** | [0.864-0.992] | 0.029 |
| **Other** | **1.029** | [0.971-1.091] | 0.331 | **0.876** | [0.833-0.921] | 0.000 | **1.073** | [0.940-1.225] | 0.295 |
| Year |  |  |  |  |  |  |  |  |  |
| *Reference: 2015* |  |  |  |  |  |  |  |  |  |
| 2016 | 0.829 | [0.771-0.891] | 0.000 | 0.961 | [0.915-1.010] | 0.051 | 1.044 | [1.009-1.082] | 0.014 |
| 2017 | 0.933 | [0.868-1.002] | 0.056 | 0.949 | [0.901-1.000] | 0.056 | 1.154 | [1.027-1.278] | 0.015 |
| 2018 | 0.617 | [0.566-0.672] | 0.000 | 0.710 | [0.673-0.749] | 0.000 | 0.966 | [0.865-1.004] | 0.064 |
| 2019 | 0.586 | [0.515-0.667] | 0.000 | 0.623 | [0.543-0.715] | 0.000 | 0.761 | [0.686-0.780] | 0.000 |
| **Campaign characteristics** |  |  |  |  |  |  |  |  |  |
| Social media shares |  |  |  |  |  |  |  |  |  |
| SMS in 1,000 | 3.130 | [2.940-3.333] | 0.000 | 1.250 | [1.169-1.337] | 0.000 | 0.848 | [0.791-0.909] | 0.000 |
| SMS2 in 1,000 | 0.970 | [0.967-0.972] | 0.000 | 0.988 | [0.978-0.999] | 0.032 | 1.009 | [1.001-1.017] | 0.025 |
| Organizer not beneficiary | 1.456 | [1.400-1.513] | 0.000 | 1.195 | [1.152-1.240] | 0.000 | 1.004 | [0.959-1.051] | 0.862 |
| Fraud flag | 0.917 | [0.831-1.012] | 0.085 | 0.850 | [0.792-0.912] | 0.085 | 0.930 | [0.874-0.989] | 0.021 |
| Solid organ | 0.852 | [0.824-0.881] | 0.000 | 0.915 | [0.887-0.944] | 0.000 | 0.967 | [0.884-1.058] | 0.464 |
| **State characteristics** |  |  |  |  |  |  |  |  |  |
| 4Q WLA per 100,000 | 1.045 | [0.968-1.128] | 0.261 | 1.072 | [1.018-1.129] | 0.008 | 0.931 | [0.885-0.981] | 0.007 |
| 4Q Uninsurance rate per 100,000 | 0.920 | [0.861-0.984] | 0.015 | 0.924 | [0.881-0.970] | 0.001 | 1.123 | [1.005-1.255] | 0.041 |
| Medicaid expansion | 1.115 | [0.988-1.259] | 0.079 | 1.063 | [1.006-1.123] | 0.030 | 1.066 | [0.979-1.160] | 0.143 |
| *Observations (n)* | 19,421 | | | 19,421 | | | 19,421 | | |

**Supplementary Table 4. Negative binomial regression estimates - IRR for all crowdfunding outcomes - full estimates**

**(A)**

|  | **Crowdfunding outcomes** | | | | | | | | | | | |
| --- | --- | --- | --- | --- | --- | --- | --- | --- | --- | --- | --- | --- |
|  | **Amount raised** | | | | | | **Success rate** | | | | | |
|  | (1) | | (2) | | (3) | | (1) | | (2) | | (3) | |
|  | IRR | 95% CI | IRR | SE | IRR | SE | IRR | SE | IRR | SE | IRR | SE |
| **Race and ethnicity** |  |  |  |  |  |  |  |  |  |  |  |  |
| *Reference: White* |  |  |  |  |  |  |  |  |  |  |  |  |
| **Black** | **0.745** | 0.029*** | **0.825** | 0.022*** | **0.847** | 0.025*** | **0.834** | 0.019*** | **0.856** | 0.017*** | **0.878** | 0.019*** |
| **Hispanic** | **0.778** | 0.070*** | **0.811** | 0.049*** | **0.797** | 0.044*** | **0.822** | 0.014*** | **0.840** | 0.015*** | **0.824** | 0.016*** |
| **Other** | **1.035** | 0.029 | **1.051** | 0.031* | **1.029** | 0.030 | **0.879** | 0.021*** | **0.888** | 0.021*** | **0.876** | 0.022*** |
| Year |  |  |  |  |  |  |  |  |  |  |  |  |
| *Reference: 2015* |  |  |  |  |  |  |  |  |  |  |  |  |
| 2016 | 1.038 | 0.035 | 0.832 | 0.032*** | 0.829 | 0.030*** | 1.018 | 0.024 | 0.960 | 0.025 | 0.910 | 0.024 |
| 2017 | 1.046 | 0.049 | 0.933 | 0.033* | 0.933 | 0.034* | 0.964 | 0.027 | 0.947 | 0.026** | 0.949 | 0.025* |
| 2018 | 0.612 | 0.030*** | 0.619 | 0.619*** | 0.617 | 0.027*** | 0.667 | 0.018*** | 0.709 | 0.019*** | 0.710 | 0.019*** |
| 2019 | 0.497 | 0.039*** | 0.590 | 0.039*** | 0.586 | 0.039*** | 0.573 | 0.040*** | 0.623 | 0.044*** | 0.623 | 0.044*** |
| **Campaign characteristics** |  |  |  |  |  |  |  |  |  |  |  |  |
| Social media shares |  |  |  |  |  |  |  |  |  |  |  |  |
| SMS in 1,000 |  |  | 3.127 | 0.103*** | 3.130 | 0.100*** |  |  | 1.247 | 0.043*** | 1.250 | 0.043*** |
| SMS2 in 1,000 |  |  | 0.970 | 0.970*** | 0.970 | 0.001*** |  |  | 0.988 | 0.005** | 0.988 | 0.005** |
| Organizer not beneficiary |  |  | 1.472 | 0.029*** | 1.456 | 0.029*** |  |  | 1.204 | 0.022*** | 1.195 | 0.022*** |
| Fraud flag |  |  | 0.915 | 0.048* | 0.917 | 0.046* |  |  | 0.853 | 0.032*** | 0.850 | 0.031*** |
| Solid organ |  |  | 0.845 | 0.015*** | 0.852 | 0.015*** |  |  | 0.911 | 0.015*** | 0.915 | 0.015*** |
| **State characteristics** |  |  |  |  |  |  |  |  |  |  |  |  |
| 4Q WLA per 100,000 |  |  |  |  | 1.045 | 0.041 |  |  |  |  | 1.072 | 0.028*** |
| 4Q Uninsurance rate per 100,000 |  |  |  |  | 0.920 | 0.031** |  |  |  |  | 0.924 | 0.023*** |
| Medicaid expansion |  |  |  |  | 1.115 | 0.069* |  |  |  |  | 1.063 | 0.030** |
| *Observations (n)* | 19,421 | | 19,421 | | 19,421 | | 19,421 | | 19,421 | | 19,421 | |

(B)

|  | **Average donation** | | | | | |
| --- | --- | --- | --- | --- | --- | --- |
|  | (1) | | (2) | | (3) | |
|  | IRR | SE | IRR | SE | IRR | SE |
| **Race and ethnicity** |  |  |  |  |  |  |
| *Reference: White* |  |  |  |  |  |  |
| **Black** | **1.040** | 0.137 | **1.033** | 0.128 | **1.014** | 0.107 |
| **Hispanic** | **0.887** | 0.034*** | **0.904** | 0.034*** | **0.926** | 0.033** |
| **Other** | **1.063** | 0.082 | **1.062** | 0.076 | **1.073** | 0.072 |
| Year |  |  |  |  |  |  |
| *Reference: 2015* |  |  |  |  |  |  |
| 2016 | 1.026 | 0.018 | 1.044 | 0.018** | 1.045 | 0.018** |
| 2017 | 1.154 | 0.071** | 1.149 | 0.069** | 1.146 | 0.064** |
| 2018 | 0.966 | 0.045 | 0.935 | 0.037* | 0.932 | 0.036* |
| 2019 | 0.761 | 0.019*** | 0.732 | 0.023*** | 0.732 | 0.024*** |
| **Campaign characteristics** |  |  |  |  |  |  |
| Social media shares |  |  |  |  |  |  |
| SMS in 1,000 |  |  | 0.848 | 0.030*** | 0.848 | 0.030*** |
| SMS2 in 1,000 |  |  | 1.009 | 0.004** | 1.009 | 0.004** |
| Organizer not beneficiary |  |  | 0.998 | 0.025 | 1.004 | 0.023 |
| Fraud flag |  |  | 0.926 | 0.027*** | 0.93 | 0.029** |
| Solid organ |  |  | 0.969 | 0.046 | 0.967 | 0.044 |
| **State characteristics** |  |  |  |  |  |  |
| 4Q WLA per 100,000 |  |  |  |  | 0.932 | 0.024*** |
| 4Q Uninsurance rate per 100,000 |  |  |  |  | 1.123 | 0.064** |
| Medicaid expansion |  |  |  |  | 1.066 | 1.066 |
| *Observations (n)* | 19,421 | | 19,421 | | 19,421 | |

**Supplementary Table 5. Negative binomial regression estimates - IRR for all crowdfunding outcomes – 2015-2018**

|  | **Crowdfunding outcomes** | | | | | |
| --- | --- | --- | --- | --- | --- | --- |
|  | **Amount raised** | | **Success rate** | | **Average donation** | |
|  | IRR | SE | IRR | SE | IRR | SE |
| **Race and ethnicity** |  |  |  |  |  |  |
| *Reference: White* |  |  |  |  |  |  |
| **Black** | **0.855** | 0.026*** | **0.883** | 0.019*** | **1.019** | 0.111 |
| **Hispanic** | **0.787** | 0.040*** | **0.819** | 0.018*** | **0.925** | 0.035** |
| **Other** | **1.026** | 0.029 | **0.885** | 0.022*** | **1.075** | 0.078 |
| Year |  |  |  |  |  |  |
| *Reference: 2015* |  |  |  |  |  |  |
| 2016 | 0.834 | 0.030*** | 0.964 | 0.024 | 1.047 | 0.019** |
| 2017 | 0.936 | 0.034* | 0.950 | 0.025* | 1.147 | 0.063** |
| 2018 | 0.617 | 0.027*** | 0.707 | 0.019*** | 0.931 | 0.035* |
|  |  |  |  |  |  |  |
| **Campaign characteristics** |  |  |  |  |  |  |
| Social media shares |  |  |  |  |  |  |
| SMS in 1,000 | 3.023 | 0.100*** | 1.232 | 0.039*** | 0.844 | 0.031*** |
| SMS2 in 1,000 | 0.971 | 0.001*** | 0.989 | 0.005** | 1.009 | 0.004** |
| Organizer not beneficiary | 1.439 | 0.029*** | 1.188 | 0.023*** | 0.996 | 0.025 |
| Fraud flag | 0.923 | 0.043* | 0.857 | 0.032*** | 0.924 | 0.029** |
| Solid organ | 0.849 | 0.015*** | 0.918 | 0.015*** | 0.965 | 0.046 |
| **State characteristics** |  |  |  |  |  |  |
| 4Q WLA per 100,000 | 1.048 | 0.040 | 1.084 | 0.030*** | 0.930 | 0.027** |
| 4Q Uninsurance rate per 100,000 | 0.926 | 0.032** | 0.922 | 0.023*** | 1.132 | 0.067** |
| Medicaid expansion | 1.113 | 0.067* | 1.055 | 0.031** | 1.073 | 0.048 |
| *Observations (n)* | 18,321 | | 18,321 | | 18,321 | |

**Supplementary Table 6. Negative binomial regression estimates - IRR for all crowdfunding outcomes – solid organs**

|  | **Crowdfunding outcomes** | | | | | |
| --- | --- | --- | --- | --- | --- | --- |
|  | **Amount raised** | | **Success rate** | | **Average donation** | |
|  | IRR | SE | IRR | SE | IRR | SE |
| **Race and ethnicity** |  |  |  |  |  |  |
| *Reference: White* |  |  |  |  |  |  |
| **Black** | **0.825** | 0.031*** | **0.878** | 0.025*** | **1.078** | 0.164 |
| **Hispanic** | **0.817** | 0.048*** | **0.853** | 0.021*** | **0.909** | 0.020*** |
| **Other** | **1.040** | 0.039 | **0.900** | 0.034*** | **0.977** | 0.028 |
| Year |  |  |  |  |  |  |
| *Reference: 2015* |  |  |  |  |  |  |
| 2016 | 0.834 | 0.029*** | 0.979 | 0.028 | 1.044 | 0.022** |
| 2017 | 0.919 | 0.034** | 0.923 | 0.030** | 1.174 | 0.104* |
| 2018 | 0.576 | 0.023*** | 0.684 | 0.022*** | 0.881 | 0.045** |
| 2019 | 0.573 | 0.038*** | 0.580 | 0.037*** | 0.721 | 0.026*** |
| **Campaign characteristics** |  |  |  |  |  |  |
| Social media shares |  |  |  |  |  |  |
| SMS in 1,000 | 3.349 | 0.100*** | 1.247 | 0.056*** | 0.816 | 0.031*** |
| SMS2 in 1,000 | 0.961 | 0.001*** | 0.989 | 0.007** | 1.013 | 0.004** |
| Organizer not beneficiary | 1.436 | 0.029*** | 1.173 | 0.029*** | 0.998 | 0.025 |
| Fraud flag | 0.938 | 0.043* | 0.866 | 0.038*** | 0.976 | 0.029** |
|  |  |  |  |  |  |  |
| **State characteristics** |  |  |  |  |  |  |
| 4Q WLA per 100,000 | 1.023 | 0.044 | 1.065 | 0.033** | 0.960 | 0.029 |
| 4Q Uninsurance rate per 100,000 | 0.914 | 0.042* | 0.914 | 0.025*** | 1.143 | 0.069** |
| Medicaid expansion | 1.081 | 0.070 | 1.054 | 0.032* | 1.026 | 0.052 |
| *Observations (n)* | 11,822 | | 11,822 | | 11,822 | |

**Supplementary Table 7. Negative binomial regression estimates - IRR for all crowdfunding outcomes – campaigns with the same race/ethnicity classification across 2 classification procedures**

|  | **Crowdfunding outcomes** | | | | | |
| --- | --- | --- | --- | --- | --- | --- |
|  | **Amount raised** | | **Success rate** | | **Average donation** | |
|  | IRR | SE | IRR | SE | IRR | SE |
| **Race and ethnicity** |  |  |  |  |  |  |
| *Reference: White* |  |  |  |  |  |  |
| **Black** | **0.864** | 0.038*** | **0.868** | 0.022*** | **1.057** | 0.125 |
| **Hispanic** | **0.834** | 0.041*** | **0.837** | 0.019*** | **0.928** | 0.027** |
| **Other** | **1.067** | 0.044 | **0.867** | 0.022*** | **1.098** | 0.079 |
| Year |  |  |  |  |  |  |
| *Reference: 2015* |  |  |  |  |  |  |
| 2016 | 0.825 | 0.030*** | 0.961 | 0.023* | 1.039 | 0.019** |
| 2017 | 0.929 | 0.030** | 0.948 | 0.026* | 1.149 | 0.065** |
| 2018 | 0.616 | 0.025*** | 0.712 | 0.020*** | 0.932 | 0.038* |
| 2019 | 0.583 | 0.036*** | 0.632 | 0.045*** | 0.727 | 0.024*** |
| **Campaign characteristics** |  |  |  |  |  |  |
| Social media shares |  |  |  |  |  |  |
| SMS in 1,000 | 3.101 | 0.100*** | 1.251 | 0.044*** | 0.847 | 0.030*** |
| SMS2 in 1,000 | 0.970 | 0.001*** | 0.988 | 0.006** | 1.009 | 0.004** |
| Organizer not beneficiary | 1.448 | 0.030*** | 1.191 | 0.024*** | 1.003 | 0.025 |
| Fraud flag | 0.911 | 0.046* | 0.851 | 0.033*** | 0.930 | 0.029** |
| Solid organ | 0.854 | 0.015*** | 0.914 | 0.016*** | 0.956 | 0.045 |
| **State characteristics** |  |  |  |  |  |  |
| 4Q WLA per 100,000 | 1.120 | 0.061** | 1.000 | 0.028 | 1.066 | 0.059 |
| 4Q Uninsurance rate per 100,000 | 0.910 | 0.032*** | 0.924 | 0.026*** | 1.102 | 0.052** |
| Medicaid expansion | 1.105 | 0.061* | 1.074 | 0.032** | 1.044 | 0.038 |
| *Observations (n)* | 18,905 | | 18,905 | | 18,905 | |

**Supplementary Table 8. Negative binomial regression estimates - IRR for all crowdfunding outcomes – campaigns with the same race/ethnicity classification across 2 classification procedures and higher certainty threshold (pr>0.6)**

|  | **Crowdfunding outcomes** | | | | | |
| --- | --- | --- | --- | --- | --- | --- |
|  | **Amount raised** | | **Success rate** | | **Average donation** | |
|  | IRR | SE | IRR | SE | IRR | SE |
| **Race and ethnicity** |  |  |  |  |  |  |
| *Reference: White* |  |  |  |  |  |  |
| **Black** | **0.799** | 0.042*** | **0.815** | 0.023*** | **1.114** | 0.201 |
| **Hispanic** | **0.787** | 0.037*** | **0.819** | 0.020*** | **0.922** | 0.030** |
| **Other** | **1.089** | 0.050* | **0.848** | 0.025*** | **1.196** | 0.175 |
| Year |  |  |  |  |  |  |
| *Reference: 2015* |  |  |  |  |  |  |
| 2016 | 0.838 | 0.029*** | 0.985 | 0.029 | 1.034 | 0.025 |
| 2017 | 0.949 | 0.032 | 0.968 | 0.031 | 1.170 | 0.090** |
| 2018 | 0.629 | 0.026*** | 0.718 | 0.028*** | 0.960 | 0.051* |
| 2019 | 0.623 | 0.044*** | 0.625 | 0.046*** | 0.734 | 0.032*** |
| **Campaign characteristics** |  |  |  |  |  |  |
| Social media shares |  |  |  |  |  |  |
| SMS in 1,000 | 3.073 | 0.095*** | 1.220 | 0.044*** | 0.847 | 0.038*** |
| SMS2 in 1,000 | 0.970 | 0.001*** | 0.991 | 0.005* | 1.009 | 0.005* |
| Organizer not beneficiary | 1.434 | 0.038*** | 1.178 | 0.024*** | 1.003 | 0.034 |
| Fraud flag | 0.896 | 0.061 | 0.829 | 0.038*** | 0.930 | 0.031*** |
| Solid organ | 0.874 | 0.016*** | 0.909 | 0.019*** | 0.956 | 0.061 |
| **State characteristics** |  |  |  |  |  |  |
| 4Q WLA per 100,000 | 1.137 | 0.069** | 0.986 | 0.033 | 1.066 | 0.079 |
| 4Q Uninsurance rate per 100,000 | 0.916 | 0.041** | 0.974 | 0.035 | 1.102 | 0.061** |
| Medicaid expansion | 1.092 | 0.066 | 1.109 | 0.037*** | 1.044 | 0.046 |
| *Observations (n)* | 12,445 | | 12,445 | | 12,445 | |

**Supplementary Analysis: using an alternative classification procedure, using the maximum probability assigned to a race/ethnicity to classify campaigns with more than one assigned race.**

**Supplementary Figure 3. Distribution of assigned probabilities by race and ethnicity categories.**

**Supplementary Table 9. Campaigns and campaign outcomes by race/ethnicity using the alternative classification procedure.**

|  | **Overall** | | **White** | | **Black** | | **Hispanic** | | **Other** | | **Test** |
| --- | --- | --- | --- | --- | --- | --- | --- | --- | --- | --- | --- |
|  |  |  | N | % | N | % | N | % | N | % | **p-value** |
| Campaigns (n) | 19,711 | | 10,474 | **53%** | 3,226 | **16%** | 2,943 | **15%** | 3,068 | **16%** |  |
|  |  |  |  |  |  |  |  |  |  |  |  |
| **Crowdfunding outcomes** | **Median** | **IQR** | **Median** | **IQR** | **Median** | **IQR** | **Median** | **IQR** | **Median** | **IQR** |  |
| Amount raised ($) | **2,770** | 1,270-6,030 | **3,040** | 1,520-6,460 | **2,323** | 870-4,950 | **2,943** | 1,025-5,360 | **2,803** | 1,235-6,365 | p < 0.001 |
| Number of donations | **32** | 15-64 | **34** | 16-66 | **29** | 12-56 | **31** | 14-60 | **33** | 14-68 | p = 0.002 |
| Monetary goal ($) | **10,000** | 5,000-25,000 | **10,000** | 5,000-25,000 | **10,000** | 5,000-25,000 | **10,000** | 5,000-25,000 | **10,000** | 5,000-30,000 | p < 0.001 |
| Success rate | **27.1%** | 9-59% | **31.0%** | 11-64% | **22.3%** | 7-52% | **23.0%** | 7-52% | **24.1%** | 7-56% | p < 0.001 |
| Average donation ($) | **81.9** | 59-113 | **85.8** | 63 -118 | **75.0** | 53-106 | **75.0** | 54-104 | **81.7** | 58-113 | p = 0.36 |

**Supplementary Figure 4. Campaigns and and WLA per 100,000 by state and race/ethnicity, using the alternative classification procedure.**

**Supplementary Figure 5. Campaigns per 100,000 and uninsurance rate, by state and race/ethnicity, using the alternative classification procedure**

**Supplementary Figure 6. Estimates of race and ethnicity adjusted IRR for each crowdfunding outcome using the alternative classification procedure**


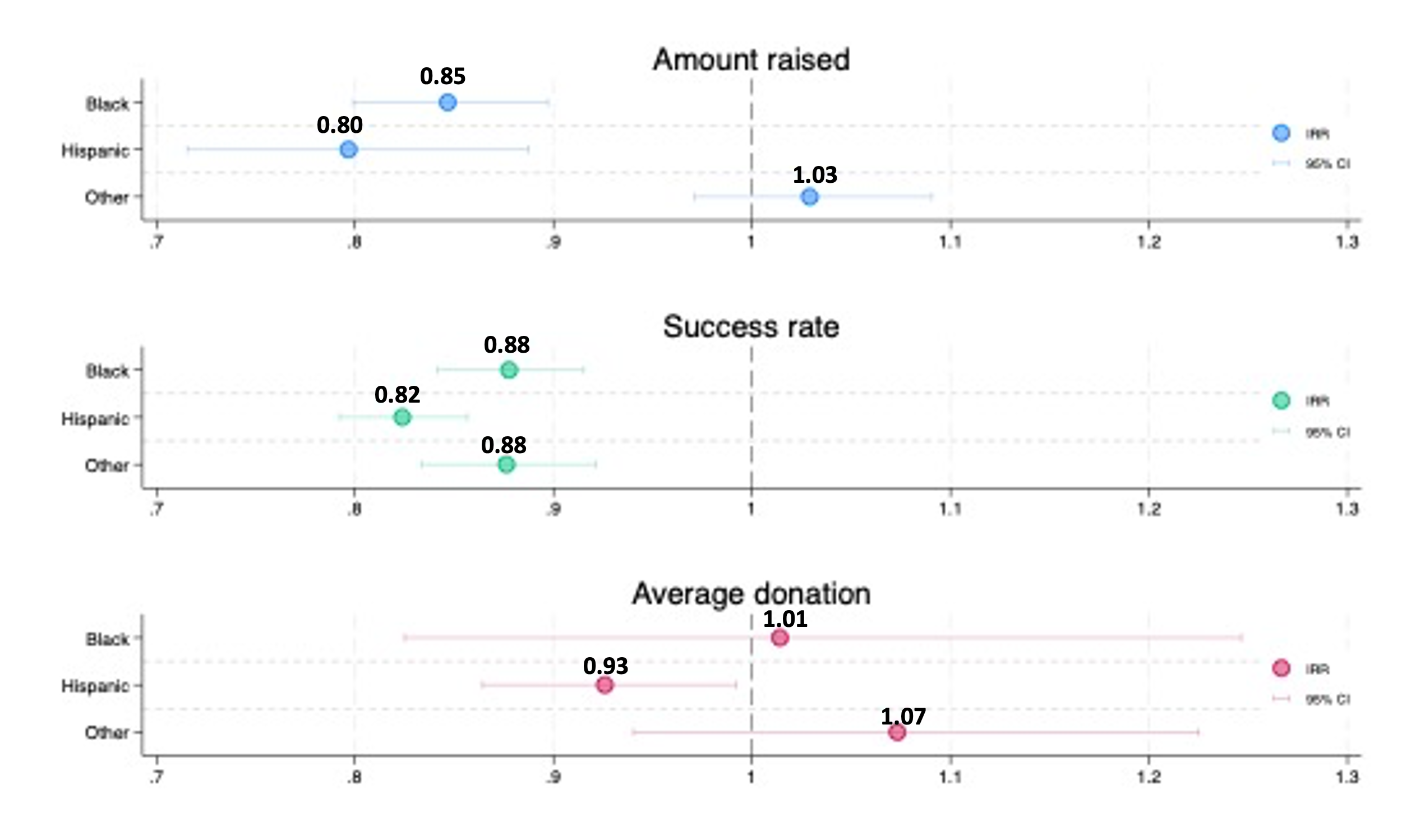


**References**

1. Perez B, Machado S, Andrews J, Kourtellis N. I call BS: Fraud Detection in Crowdfunding Campaigns. In: ACM; 2022. doi:10.1145/3501247.3531541

2. Namsor | Name checker for Gender, Origin and Ethnicity determination. Accessed September 7, 2023. https://namsor.app/

3. Sebo P. Performance of gender detection tools: a comparative study of name-to-gender inference services. *J Med Libr Assoc*. 109(3):414-421. doi:10.5195/jmla.2021.1185

4. US Cities List. Published online 2019. https://www.uscitieslist.org/
